# Distribution of alien plant species of the Middle Volga Region (South-East of the European part of Russia): a dataset

**DOI:** 10.3897/BDJ.8.e59125

**Published:** 2020-11-09

**Authors:** Stepan Senator, Alyona Tretyakova, Dmitry Vorontsov

**Affiliations:** 1 Tsitsin Main Botanical Garden of the Russian Academy of Sciences, Moscow, Russia Tsitsin Main Botanical Garden of the Russian Academy of Sciences Moscow Russia; 2 Samara Federal Research Center of the Russian Academy of Sciences, Institute of Ecology of the Volga River Basin of the Russian Academy of Science, Togliatti, Russia Samara Federal Research Center of the Russian Academy of Sciences, Institute of Ecology of the Volga River Basin of the Russian Academy of Science Togliatti Russia; 3 Botanical Garden of Ural branch of the Russian Academy of Sciences, Ekaterinburg, Russia Botanical Garden of Ural branch of the Russian Academy of Sciences Ekaterinburg Russia; 4 Ural Federal University named after the first President of Russia B.N.Yeltsin, Ekaterinburg, Russia Ural Federal University named after the first President of Russia B.N.Yeltsin Ekaterinburg Russia; 5 The Russian State University of Justice, Moscow, Russia The Russian State University of Justice Moscow Russia

**Keywords:** dataset, vascular plants, alien and invasive species, literary sources, ﬁeld study, data paper, European part of Russia

## Abstract

**Background:**

The dataset presented in the current study contains information regarding alien vascular plant species found in the Middle Volga Region (South-East of the European part of Russia). The dataset overall includes 413 species belonging to 247 genera and 67 families. The described dataset is based on the data published during floristic studies from 1851 to 2019. The dataset does not include alien vascular plant species that have presently disappeared from the territory of the region. It contains a total of 7,782 records of occurrences, extracted from the *Salix* system of information and analytics, developed in the Institute of Ecology of the Volga River Basin of the Russian Academy of Sciences.

**New information:**

A total of 7,782 records were published on the occurrence of alien vascular plants in the Middle Volga Region. Each entry includes information regarding the place of occurrence of the alien plant species, the year of occurrence, the person who recorded the alien plant and who identified it, status of the species (introduced or invasive), link to the herbarium, which contains the specimen and the literary source. If it were impossible to establish the names of the persons who collected the samples and (or) their identification in the identifiedBy and recordedBy fields, the names of the authors of the publication given in the associatedReferences field were entered. The presented dataset supplements the information on the distribution of alien plant species in the whole European part of Russia and specifies the places of their findings in the Middle Volga Region.

## Introduction

Currently, the invasion of alien plant species constitutes a large-scale phenomenon that poses a threat to the natural diversity and is regarded as one of the most serious environmental problems that mankind faces today (Lambdon et al. 2008, Vinogradova et al. 2010, Pyšek et al. 2017, Senator and Rozenberg 2017). The Middle Volga Region is one of the leading regions of Russia in terms of industrial development and economic infrastructure. The area of the Region is 99,000 km^2^; the population density is 48.6 per km^2^. The position of the Middle Volga Region, situated in the intersection of large transport routes of both latitudinal and longitudinal directions, and the significant population density are determining factors for the richness of alien flora and the high rates of its dynamics (Rozenberg 2009). This article contains the dataset records on alien vascular plant species occurrences, published in GBIF as a Darwin Core Archive (Senator 2020), prepared in accordance with the concept of "data paper" (Penev et al. 2017).

## General description

### Purpose

The main purpose of this study is the presentation of a published dataset on the distribution and the composition of alien plant species of the Middle Volga Region (South-East of the European part of Russia) in the GBIF as a Darwin Core Archive. In addition, the study aims to provide accurate information on the global distribution of alien plant species, based on the records of their growth outside of their native ranges and enables the creation of large-scale plant invasion models.

## Project description

### Title

№ 17-117112040040-3 «Assessment of modern biodiversity and forecast of its change for the ecosystems of the Volga River Basin in the conditions of their natural and anthropogenic transformation»

№ 18-118021490111-5 «Biological diversity of natural and cultural flora: fundamental and applied research and conservation issues»

### Personnel

Stepan Senator, Alyona Tretyakova, Dmitry Vorontsov

### Study area description

**Figure 1.**
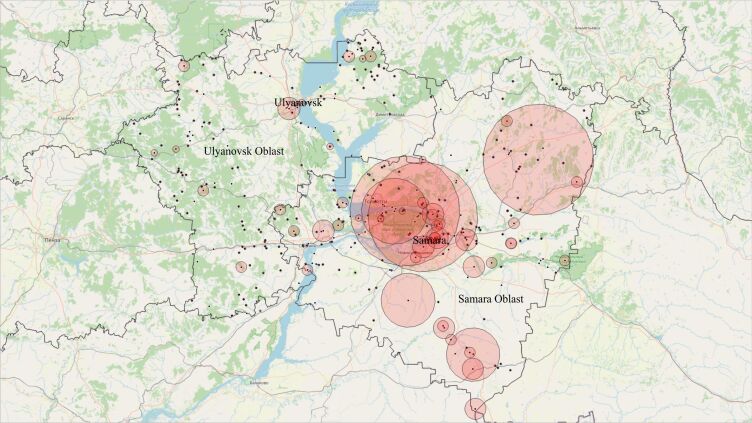
Alien plant species occurrences points (black dots) and coordinate uncertainty zones (red semi-transparent circles) in the study of the Middle Volga Region.

The Middle Volga Region is located in the South-East of the European part of Russia. From the aspect of Regional Administration, it is considered as Samara and Ulyanovsk Oblasts of the Russian Federation (Fig. 1).

### Design description

The first step of the study was to create a checklist of alien plant species found in the Middle Volga Region (Senator and Vasjukov 2019). The conceptual basis of the study is the adoption of the idea of the invasive species as an alien plant naturalising into the natural and semi-natural plant communities (Richardson et al. 2010a, Baranova et al. 2018). Hence, all other alien plant species are either recognised as potentially invasive (established and successfully propagating in places where they are introduced, but not spreading into natural and semi-natural plant communities) or non-naturalised.

The dataset is based on Samara and Ulyanovsk Oblasts’ published data on the flora, which are confirmed through stored samples in Herbaria of the Lomonosov Moscow State University (MW), V.L. Komarov Botanical Institute of the Russian Academy of Sciences (LE), Institute of Ecology of the Volga River Basin of the Russian Academy of Sciences (PVB), Ulyanovsk State Pedagogical University named after I.N. Ulyanov (UPSU), Mordovian State University (GMU), Samara State University (SMR) and Moscow Pedagogical State University (MOSP). Additional field research materials, conducted by specialists from the Institute of Ecology of the Volga River Basin of the Russian Academy of Sciences, were taken into account. The listed data are stored in the *Salix* system of information and analytics, developed in the Institute of Ecology of the Volga River Basin of the Russian Academy of Sciences (Senator et al. 2018).

The earliest data included, regarding the growth of alien plant species in the territory of the indicated region, dates from 1851 to 2019 as the most recent.

### Funding

This work was supported by the Competitiveness of the Ural Federal University (Russian Federation Government Regulation no. 211, contract no. 02. A03.21.0006).

## Sampling methods

### Study extent

The presented dataset is based on the published synopsis of alien plants species of the Middle Volga Region (Senator and Vasjukov 2019) and materials stored in the *Salix* system of information and analytics (Senator et al. 2018). The dataset does not include alien vascular plant species that have presently disappeared from the territory of the Region, likewise species that are native in one part of the study Region, while in another they are found exclusively in disturbed habitats. The terms "introduced species" and "invasive species" are used in accordance with Richardson et al. (2010b) and the system of terms used in Russian-language scientific literature (Baranova et al. 2018).

### Sampling description

In 2017, the Salix database was created in order to store floristic information for the Middle Volga Region. The database contains records of all plant species growing in the area of the study. Each entry contains a link to a literary source or a herbarium specimen confirming the findings. The publication of the synopsis of alien plant species in the Middle Volga Region (Senator and Vasjukov 2019) made it possible to highlight and identify the alien plant species. The information about the collectors of herbarium specimens is presented in Table 1.

There are 1472 occurrences in the dataset with coordinate uncertainty of 100 km. This is due to the fact that, in the literary sources of the late 19th and early 20th centuries, there is no exact geolocation. Instead, it is specified, for example, "Western part of Buguruslansky Uyezd" or "neighbourhood of Sergievsk". This collection could be made within a radius of 100 km or more from a settlement. At the same time, such data are of interest from the point of view of identifying the dynamics of the appearance of alien species in the study area.

### Quality control

During the development process of the dataset, the records obtained from primary sources were examined by the first author, as some names of the species did not correspond to the modern nomenclature. The herbarium samples were collected and identified by scientists from the Institute of Ecology of the Volga River Basin of the Russian Academy of Sciences and are stored in the PVB herbarium. The accuracy of the determination of some samples was confirmed by taxonomists from the Lomonosov Moscow State University (MW) and V.L. Komarov Botanical Institute of the Russian Academy of Sciences (LE). For the purposes of publishing the dataset in the GBIF network, the records have been adjusted to match the Darwin Core specifications (Wieczorek et al. 2012).

### Step description

1. The initial data for this standardised dataset are the information stored in the *Salix* system of information and analytics, available at https://саликс.рф/Salix/. This information is updated regularly.

2. The *Salix* system of information and analytics enables us to generate a report in an Excel table form with the necessary dataset. For the purposes of the report, the fields «Species name», «Family», «Administrative Oblast», «District», «Location», «Geographical coordinates», unique identifiers for each taxon and bibliographic references were generated, containing the necessary information regarding the findings. In cases of an absence of a reference, a herbarium sample collector is specified.

3. The scientific names of plants were adjusted in accordance with the GBIF Taxonomic Backbone.

The dataset fields’ names were chosen according to Darwin Core (Wieczorek et al., 2012) and include the following: "scientificName", "taxonRank", "kingdom", "phylum", "class", "family", "genus", "specificEpithet", "infraspecificEpithet", "stateProvince", "municipality", "locality", "decimalLatitude", "decimalLongitude", "coordinateUncertaintyInMetres", "georeferencedBy", "establishmentMeans", "year", "recordedBy", "identifiedBy", "сollectionCode", "geodeticDatum", "basisOfRecord", "countryCode", "country", "occurrenceID", "institutionCode", "language", "associatedReferences".

## Geographic coverage

### Description

The Middle Volga Region is a region located in the South-East of the European part of Russia. In botanical-geographical terms, it is located on the border of the European broad-leaved forest area and the Eurasian steppe area. The area of the region is 99,000 km^2^; the population density is 48.6 per km^2^. From the aspect of Regional Administration, it is considered as Samara and Ulyanovsk Oblasts of the Russian Federation. The coordinates of the northernmost points are 54.887903 N, 49.289177 E, the southernmost points are 51.774088 N, 50.766883 E, the Western points are 53.988244 N, 45.797979 E and the Eastern points are 54.340690 N, 52.566412 E.

The climatic conditions of the Middle Volga Region are formed under the influence of the drifting western Atlantic Ocean air masses and the Siberian anticyclone macrocirculations. The climate is temperate-continental, with peculiar seasonal circulation, characterised by sharp temperature contrasts between cold and warm seasons, a rapid transition from cold winter to hot summer, moisture deficiency, high evaporation rate and abundance of sunlight. The continentality of the climate increases from west to east. The average January temperature is -11 – -13.5°С, in July – +19°С – +21.0°С. The frost-free period lasts 118–157 days. On the territory of the Region, there is a zonal decrease in annual precipitation from north to south from 540 to 370 mm. The largest part of the area of study is located in the forest-steppe zone, characterised by the presence of typical chernozem soil, an alternation of broad-leaved forests and a rich variety of steppe grasslands. The extreme south point of the territory is occupied by a steppe zone with forb-fescue-feather grass steppes on ordinary and southern chernozem soils.

The largest number of the records was made in the Ulyanovsk Oblast (4052 records or 52%), the smallest in the Samara Oblast (3730 records or 48%).

### Coordinates

51.774 and 54.888 Latitude; 45.798 and 52.566 Longitude.

## Taxonomic coverage

### Description

The dataset includes records of expansion on alien plant species in two groups (Pinophyta and Magnoliophyta), 67 families, 247 genera and 413 species (Senator 2020). The majority of the records is occupied by the Magnoliophyta group. The highest number of the records refer to the Dicotyledones (90%), followed by the Monocotyledones (9.7%). The Pinophyta group is represented by a single family of Pinaceae, one genus and one species *Larix sibirica* (0.3% of records).

The Asteraceae, Poaceae, Brassicaceae, Rosaceae, Fabaceae, Amaranthaceae, Lamiaceae, Solanaceae, Caryophyllaceae and Apiaceae families have a wide range of multi-species and hold the maximum number of records, totalling 255 species that comprise 67% of the records (Table 2). These families are characteristic of European floras’ top spectrum (Lambdon et al. 2008).

A total of 18 families contain a small number of records (less than 10). In this case, the families Cleomaceae, Commelinaceae, Linaceae and Verbenaceae are represented by only one species and one occurrence.

**Figure 2.**
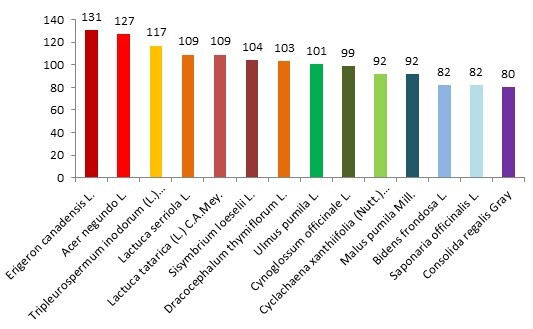
The species with the largest number of occurrences (species with more than 900 records are shown).

The obtained data provided the proper grounds for identifying the most common alien plant species in the Middle Volga Region. Amongst them are *Erigeron canadensis* L., *Acer negundo* L., *Tripleurospermum inodorum* (L.) Sch.Bip., *Lactuca serriola* L., *Lactuca tatarica* (L.) C.A.Mey. etc. (Fig. 2)

## Temporal coverage

### Notes

1851 – 2019

**Figure 3.**
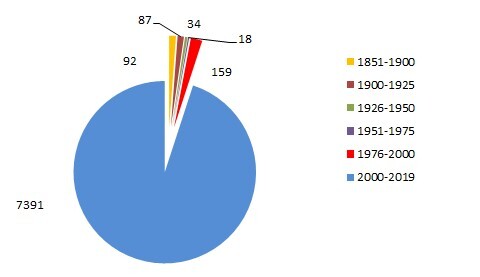
Number of records through different years of observation.

The presented dataset contains information on the occurrences of alien plants since 1851, with the most recent findings recorded in 2019. Fig. 3 shows that, in the 19th century and in early 20th century, the number of findings of alien plant species in that period was small. The number of occurrences increases by the end of the 20th century, with the largest number of records registered between 2000 and 2019. This result is connected with the growing interest in the study of alien plant species and the active work conducted by the staff of the Institute of Ecology of the Volga Basin of the Russian Academy of Sciences, in particular S.V. Saksonov, N.S. Rakov, S.A. Senator, V.M. Vasyukov etc.

## Usage licence

### Usage licence

Other

### IP rights notes

This work is licensed under a Creative Commons Attribution (CC-BY) 4.0 Licence.

## Data resources

### Data package title

Alien plant species of the Middle Volga Region (South-East of the European part of Russia)

### Resource link

https://www.gbif.org/ru/dataset/0777230d-c252-4f99-9c16-cc63c09dfd52

### Alternative identifiers

https://doi.org/10.15468/gv7hxs

### Number of data sets

1

### Data set 1.

#### Data set name

Alien plant species of the Middle Volga Region (South-East of the European part of Russia)

#### Data format

Darwin Core

#### Number of columns

29

#### Data format version

1.6

#### Description

The presented dataset (Senator 2020) contains information about alien vascular plant species found in the Middle Volga Region (South-East of the European part of Russia). In total, this dataset includes 413 species belonging to 247 genera and 67 families. The study is based on data published during floristic studies from 1851 to 2019. At the same time, the study does not include alien vascular plant species that have now disappeared from the territory of the Region. In total, the dataset contains 7782 occurrence records which were extracted from the *Salix* information and analytical system developed at the Institute of Ecology of the Volga River Basin of the Russian Academy of Sciences (Senator et al. 2018).

**Data set 1.** 

Column label	Column description
scientificName	The full scientific name. http://rs.tdwg.org/dwc/terms/scientificName
taxonRank	The taxonomic rank of the most specific name in the scientificName. http://rs.tdwg.org/dwc/terms/taxonRank
kingdom	The full scientific name of the kingdom in which the taxon is classified. http://rs.tdwg.org/dwc/terms/kingdom
phylum	The full scientific name of the phylum or division in which the taxon is classified. http://rs.tdwg.org/dwc/terms/phylum
class	The full scientific name of the class in which the taxon is classified. http://rs.tdwg.org/dwc/terms/class
family	The full scientific name of the family in which the taxon is classified. http://rs.tdwg.org/dwc/terms/family
genus	The full scientific name of the genus in which the taxon is classified. http://rs.tdwg.org/dwc/terms/genus
specificEpithet	The name of the first or species epithet of the scientificName. http://rs.tdwg.org/dwc/terms/specificEpithet
infraspecificEpithet	The name of the lowest or terminal infraspecific epithet of the scientificName, excluding any rank designation. http://rs.tdwg.org/dwc/terms/infraspecificEpithet
stateProvince	The name of the next smaller administrative region than country. http://rs.tdwg.org/dwc/terms/stateProvince
municipality	The full, unabbreviated name of the next smaller administrative region than county. http://rs.tdwg.org/dwc/terms/municipality
locality	The specific description of the place. Less specific geographic information can be provided in other geographic terms (higherGeography, continent, country, stateProvince, county, municipality, waterBody, island, islandGroup). This term may contain information modified from the original to correct perceived errors or standardise the description. http://rs.tdwg.org/dwc/terms/locality
decimalLatitude	The geographic latitude (in decimal degrees, using the spatial reference system given in geodeticDatum) of the geographic centre of a Location. http://rs.tdwg.org/dwc/terms/decimalLatitude
decimalLongitude	The geographic longitude (in decimal degrees, using the spatial reference system given in geodeticDatum) of the geographic centre of a Location. http://rs.tdwg.org/dwc/terms/decimalLongitude
coordinateUncertaintyInMetres	The horizontal distance (in metres) from the given decimalLatitude and decimalLongitude describing the smallest circle containing the whole of the Location. Leave the value empty if the uncertainty is unknown, cannot be estimated or is not applicable (because there are no coordinates). Zero is not a valid value for this term. http://rs.tdwg.org/dwc/terms/coordinateUncertaintyInMeters
georeferencedBy	A list (concatenated and separated) of names of people, groups or organisations who determined the georeference (spatial representation) for the Location. http://rs.tdwg.org/dwc/terms/georeferencedBy
establishmentMeans	The process by which the biological individual(s) represented in the Occurrence became established at the location. http://rs.tdwg.org/dwc/terms/establishmentMeans
year	The four-digit year in which the Event occurred, according to the Common Era Calendar. http://rs.tdwg.org/dwc/terms/year
recordedBy	A list (concatenated and separated) of names of people, groups or organisations responsible for recording the original Occurrence. http://rs.tdwg.org/dwc/terms/recordedBy
identifiedBy	A list (concatenated and separated) of names of people, groups or organisations who assigned the Taxon to the subject. http://rs.tdwg.org/dwc/terms/identifiedBy
сollectionCode	The name, acronym, coden or initialism identifying the collection or dataset from which the record was derived. http://rs.tdwg.org/dwc/terms/collectionCode
geodeticDatum	The ellipsoid, geodetic datum or spatial reference system (SRS) upon which the geographic coordinates given in decimalLatitude and decimalLongitude are based. http://rs.tdwg.org/dwc/terms/geodeticDatum
basisOfRecord	The specific nature of the data record. Included value: HumanObservation. http://rs.tdwg.org/dwc/terms/basisOfRecord
countryCode	The standard code for the country in which the Location occurs. Included value: RU. http://rs.tdwg.org/dwc/terms/countryCode
country	The name of the country or major administrative unit in which the Location occurs. Included value: Russia http://rs.tdwg.org/dwc/terms/country
occurrenceID	An identifier for the Occurrence (as opposed to a particular digital record of the occurrence). In the absence of a persistent global unique identifier, construct one from a combination of identifiers in the record that will most closely make the occurrenceID globally unique. http://rs.tdwg.org/dwc/terms/occurrenceID
institutionCode	The name (or acronym) in use by the institution having custody of the object(s) or information referred to in the record. http://rs.tdwg.org/dwc/terms/institutionCode
language	A language of the resource. Included value: ru. http://purl.org/dc/terms/language
associatedReferences	A list (concatenated and separated) of identifiers (publication, bibliographic reference, global unique identifier, URI) of literature associated with the Occurrence. http://rs.tdwg.org/dwc/terms/associatedReferences

## Figures and Tables

**Table 1.** Number of records made by authors. Only authors with more than 100 records are shown.

**Authors**	**Number of records**
Saksonov S.V.	4192
Rakov N.S.	4142
Senator S.A.	3689
Vasjukov V.M.	1876
Ivanova A.V.	1635
Dronin G.V.	758
Kornilov S.P.	718
Novikova L.A.	686
Goljusheva A.N.	558
Lashmanova N.N.	459
Sytin A.K.	389
Savenko O.V.	376
Plaksina T.I.	316
Silaeva T.B.	280
Istomina E.Yu.	261
Bobkina E.M.	244
Nikitin N.A.	227
Koneva N.V.	177
Ilina V.N.	158
Solovyova V.V.	153

**Table 2.** Taxonomic distribution of alien plant species and species entries amongst families in the dataset. Families are listed in order of decreasing total number of entries.

**Plant family**	**Number of**			% **of all occurrences**
	**genera**	**species**	**records**	
Asteraceae	34	59	1660	21.3
Poaceae	24	40	592	7.6
Brassicaceae	18	34	938	12.1
Rosaceae	15	32	431	5.5
Fabaceae	14	23	305	3.9
Amaranthaceae	11	20	488	6.3
Lamiaceae	7	13	319	4.1
Solanaceae	8	12	172	2.2
Caryophyllaceae	6	11	195	2.5
Apiaceae	11	11	127	1.6
Boraginaceae	7	9	205	2.6
Polygonaceae	5	8	116	1.5
Euphorbiaceae	2	8	28	0.4
Cucurbitaceae	6	7	133	1.7
Amaryllidaceae	2	7	50	0.6
Malvaceae	4	6	89	1.1
Plantaginaceae	3	6	51	0.7
Papaveraceae	3	6	50	0.6
Onagraceae	2	5	139	1.8
Ranunculaceae	3	5	109	1.4
Salicaceae	2	5	108	1.4
Sapindaceae	2	4	149	1.9
Oleaceae	2	4	109	1.4
Adoxaceae	2	4	84	1.1
Geraniaceae	2	4	69	0.9
Vitaceae	2	4	65	0.8
Convolvulaceae	3	4	47	0.6
Grossulariaceae	1	3	83	1.1
Violaceae	1	3	79	1.1
Caprifoliaceae	3	3	75	0.9
Berberidaceae	1	3	40	0.5
Crassulaceae	2	3	13	0.2
Apocynaceae	2	3	8	0.1
Ulmaceae	1	2	115	1.5
Elaeagnaceae	2	2	96	1.2
Urticaceae	1	2	37	0.5
Oxalidaceae	1	2	27	0.4
Balsaminaceae	1	2	23	0.3
Rubiaceae	1	2	21	0.3
Paeoniaceae	1	2	18	0.2
Polemoniaceae	2	2	12	0.2
Juncaceae	2	2	5	0.1
Araceae	2	2	3	0.1
Cannabaceae	1	1	73	0.9
Hydrocharitaceae	1	1	42	0.5
Typhaceae	1	1	32	0.4
Pinaceae	1	1	21	0.3
Portulacaceae	1	1	19	0.2
Asphodelaceae	1	1	15	0.2
Iridaceae	1	1	14	0.2
Cornaceae	1	1	13	0.2
Orobanchaceae	1	1	13	0.2
Hydrangeaceae	1	1	10	0.1
Hydrophyllaceae	1	1	8	0.1
Celastraceae	1	1	6	< 0.1
Fagaceae	1	1	6	< 0.1
Juglandaceae	1	1	6	< 0.1
Anacardiaceae	1	1	4	< 0.1
Acoraceae	1	1	3	< 0.1
Mazaceae	1	1	3	< 0.1
Tamaricaceae	1	1	3	< 0.1
Menyanthaceae	1	1	2	< 0.1
Tetradiclidaceae	1	1	2	< 0.1
Cleomaceae	1	1	1	< 0.1
Commelinaceae	1	1	1	< 0.1
Linaceae	1	1	1	< 0.1
Verbenaceae	1	1	1	< 0.1
67	248	413	7782	100
